# Extraction of terminal ileal lipomas to cecum can facilitate endoscopic resection: A case series with video

**DOI:** 10.1002/deo2.375

**Published:** 2024-04-30

**Authors:** Hiroshi Yamazaki, Yohei Minato, Deepak Madhu, Toshifumi Iida, Susumu Banjyoya, Tomoya Kimura, Koichi Furuta, Shinya Nagae, Yohei Itou, Nao Takeuchi, Shunya Takayanagi, Yoshiaki Kimoto, Yuki Kano, Takashi Sakuno, Kohei Ono, Ken Ohata

**Affiliations:** ^1^ Department of Gastrointestinal Endoscopy NTT Medical Center Tokyo Tokyo Japan

**Keywords:** endoscopic submucosal dissection, endoscopic mucosal resection, lipoma, submucosal tumor, traction

## Abstract

Large ileal lipomas over 2 cm can cause symptoms, that may require a resection. Due to the narrow lumen and thin walls of the ileum, endoscopic treatments can have a high risk of adverse events and require technical expertise, thus surgical resection is currently the mainstay of treatment. To overcome the technical challenges, we developed a novel method to endoscopically resect terminal ileal lipomas. The technique involves extracting the lesion into the cecum, which creates sufficient space to maneuver, and a better field of view. The lipoma is resected with endoscopic mucosal resection or endoscopic submucosal dissection. The appearance of the lipoma protruding out of the ileocecal valve resembles that of a tongue sticking out of the mouth, thus we named this the “tongue out technique”. To assess the technical feasibility of this method, we retrospectively analyzed seven cases of terminal ileal lipoma that were endoscopically resected using the “tongue out technique” at NTT Medical Center Tokyo between January 2017 and October 2023. Technical success was 100% and en bloc resection was achieved in all cases. The median size was 31 (14–55) mm. Three cases were resected with endoscopic mucosal resection while endoscopic submucosal dissection was performed on the other four cases. There was one case of delayed post‐endoscopic mucosal resection bleeding, which was caused by clip dislodgement. There were no perforations. No recurrence of the lipoma or associated symptoms have been observed. This new technique can allow more ileal lipomas to be treated with minimally invasive and organ‐preserving endoscopic procedures.

## INTRODUCTION

Lipoma is a subepithelial lesion that usually arises from the submucosa.^1^, ^2^ Lipomas account for 0.035%–4.4% of benign gastrointestinal lesions and can be seen throughout the gastrointestinal tract.^1^ While most lipomas are small and asymptomatic, ileal lipomas larger than 2 cm can cause abdominal symptoms such as pain, diarrhea, bleeding, and intussusception.^1^ Endoscopically, lipomas are yellowish subepithelial lesions with a positive “cushion sign”.^3^ It is seen as low attenuation mass in non‐contrast computed tomography scans.^4^ Various endoscopic methods have been proposed for the resection of colonic lipomas, but the treatment of ileal lipoma has not been established. There have been several case reports on endoscopic resection of ileal lipomas, but there have been reports of perforation during resection, as well as other reports of symptom recurrences due to incomplete resection. The high technical expertise required, and the high rate of adverse events have left surgical resection as the mainstay of treatment.^1^, ^2^ To overcome the technical challenges, we developed a novel method called the “tongue out technique” to endoscopically resect ileal lesions within the cecum, which results in better working space and maneuverability.

## PROCEDURE OR TECHNIQUE

Upon colonoscope insertion to the cecum, the terminal ileum lipoma can often be seen spontaneously protruding out of the ileocecal valve. For lesions that return into the ileum with insufflation, forceps are used to extract the lipoma out into the cecum. The extraction of the lipoma out of the ileocecal valve allows the circumferential observation of the base of the lesion to determine the resection line. Thereafter, the lipoma is resected with either endoscopic mucosal resection (EMR) or endoscopic submucosal dissection (ESD). 

EMR is performed on lesions that have relatively long stalks, allowing circumferential observation and hence, safe snaring around the base of the lesion. These lesions are mostly pedunculated; spontaneously protruding out of the ileocecal valve and often remaining within the cecum. The lesions are further pulled into the cecum for better visualization of the base. For lesions with relatively thick stalks, a detachable ligation loop is applied before resection, to prevent post‐EMR bleeding from the large vessels within the stalk.

**FIGURE 1:**
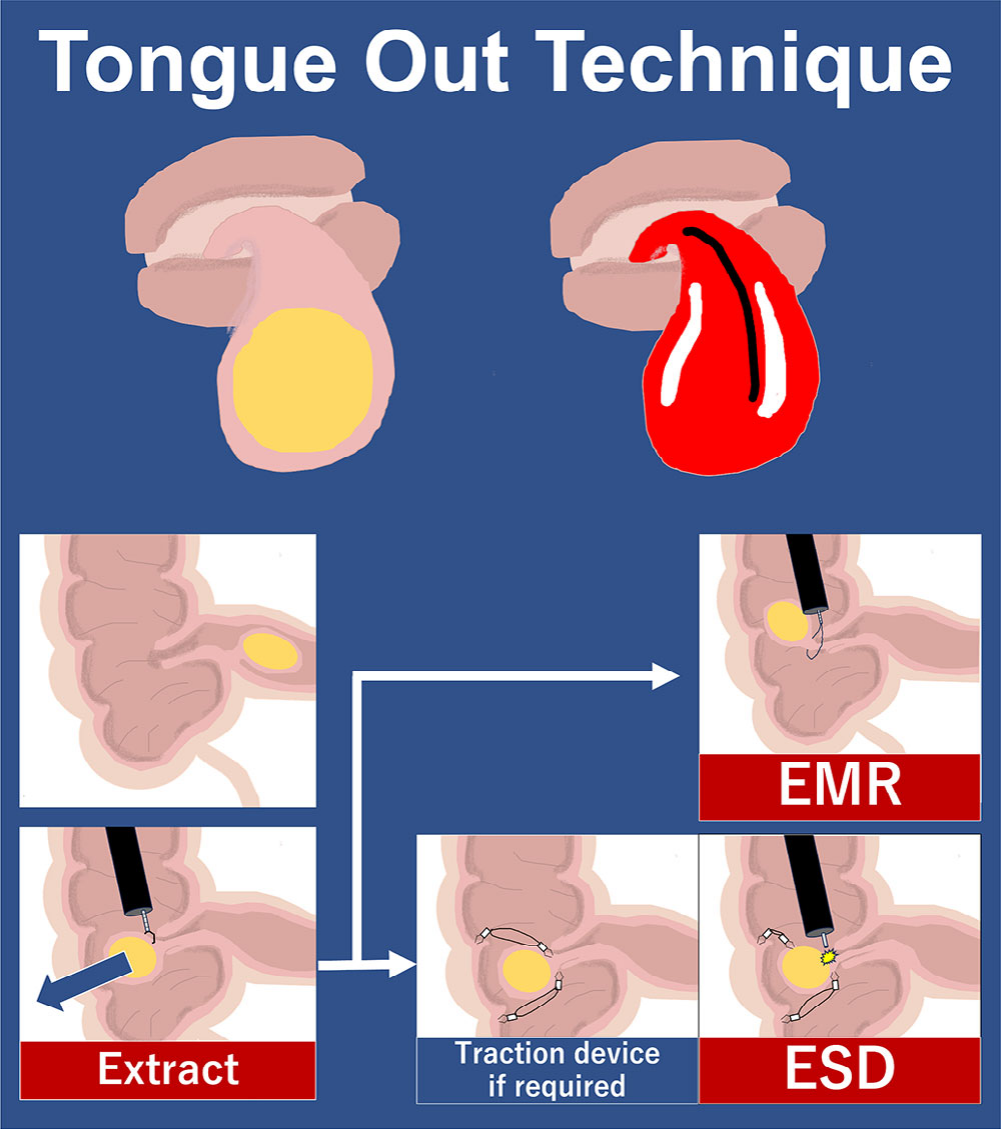
Tongue out technique involves extracting the lipoma out of the ileocecal valve. The appearance of the protruding lipoma resembles a tongue sticking out of the mouth. After extracting the lipoma, endoscopic mucosal resection (EMR) is performed on lesions where the snare could be deployed safely around the base. Endoscopic submucosal dissection (ESD) is performed on other lesions with short, wide bases. When the lipoma returns to the ileum upon insufflation, traction devices are used to secure its position.

ESD is chosen as the resection method when safe snare deployment around the base is not feasible due to the wide base of the lesion. For lesions that return into the ileum upon insufflation, a traction device is applied to secure the lesion within the cecum and ESD is performed thereafter. A hemostatic clip with a band is first attached to the base of the lesion. The other end of the band is grasped using a reopenable clip and clipped onto the cecal wall, opposite to the ileocecal valve, creating tension between the cecal wall and the lesion. The same procedure is repeated on the other side of the base for additional stability. Subsequently, mucosal incision is initiated from the oral side of the lesion, followed by submucosal dissection, using a needle‐type knife. After complete resection, the post‐ESD defect is completely closed using clips. The lipoma will remain in the cecum due to the traction band, which is later removed using the knife. A schematic diagram describing the procedure is shown below (Figure 1). The specimen is ultimately retrieved using a net retrieval device.

## RESULTS

### Patients and methods

We retrospectively analyzed seven cases of terminal ileal lipoma endoscopically resected using the “tongue out technique” at NTT Medical Center Tokyo, between January 2017 and October 2023. A total of eleven cases were pointed out during the study period. The lesions were identified using a regular colonoscope, either during an abdominal symptom evaluation or during a colonoscopy for colorectal cancer screening. After a thorough explanation of the risks and benefits of prophylactic treatment, endoscopic resection was performed when the patient preferred the treatment. Seven patients underwent endoscopic treatment, two patients were referred to another hospital due to patient preference, and one patient chose regular follow‐up. Another case was identified during an endoscopic evaluation for recurring constipation. While the patient had no abdominal pain, an abdominal computed tomography scan incidentally revealed intussusception, and after discussion with the surgeon, the patient underwent elective surgery. For the seven cases that were endoscopically treated, we assessed technical success, en bloc resection rate, procedure time, adverse event, and recurrence rate.

### Results

All endoscopically treated lesions were resected using the “tongue out technique”. Six out of seven patients were male, with a median age of 74 (range: 50–75) years. Only one case had symptoms and the rest were detected incidentally. Treatment was performed in five cases due to the large size and potential to cause symptoms, one was resected due to a possible differential diagnosis of neuroendocrine tumor, and one due to symptoms that were attributed to the lipoma. Three cases were resected by EMR and the other four cases by ESD. ESD was chosen as the treatment method when safe snare deployment around the base was not feasible due to the wide base of the lesion. Prior to EMR in two of the cases, a detachable ligation device (Endoloop; Olympus) was tightened around the base for lesions with a long stalk. All lesions were resected in the cecum, either by endoscopic extraction or by spontaneous protrusion. For three lesions that returned into the ileum upon insufflation, clip, and band traction were applied to stabilize the lesion within the cecum. Only one adverse event was noted. This was an instance of delayed post‐EMR bleeding, which was attributable to clip dislodgement. There were no perforations. Technical success was 100% (7/7). En‐bloc resection was achieved in all cases. The median duration of the admissions was 3 days for EMR cases and 5 days for ESD cases. After discharge, patients were instructed to undergo a colonoscopy follow‐up one year after the treatment, followed by regular endoscopic surveillance at the referring clinics. To this day, no patient with a recurrence has been referred to our hospital. A brief description of the cases is given in the table below (Table 1).

**TABLE 1:** Characteristics and outcomes of “tongue out technique”.

No.	Year	Age (years)	Sex	Symptoms	Comorbidity	Size (mm)	Procedure	Resection site	Device	Technical success	Procedure time (min)	En bloc resection	Adverse event	Hospital stay (days)
1	2017	74	M	None	DM	31 × 30 × 18	EMR	Cecum	Snare master	Yes	6	Yes	None	3
2	2020	74	M	Abdominal pain	BPH, DM, DL, GERD, and HT	30 × 25 × 34	ESD	Cecum	Dual Knife	Yes	40	Yes	None	9
3	2020	75	M	None	Appendicitis, CKD, and HT	37 × 32 × 30	“Loop‐and‐let‐go” + EMR	Cecum	Snare master	Yes	4	Yes	None	5
4	2021	50	M	None	None	28 × 25 × 16	ESD+traction	Cecum	Dual Knife	Yes	40	Yes	None	3
5	2022	75	M	None	DL and IPMN	14 × 9 × 7	ESD+traction	Cecum	Tech knife	Yes	10	Yes	None	5
6	2022	61	F	None	GERD and Gout	55 × 37 × 23	“Loop‐and‐let‐go” + EMR	Cecum	Dualoop	Yes	14	Yes	Delayed bleeding	5
7	2023	53	M	None	AF	34 × 33 × 14	ESD+traction	Cecum	Dual Knife	Yes	30	Yes	None	4

Abbreviations: AF, atrial fibrillation; BPH, benign prostatic hyperplasia; CKD, chronic kidney disease; DL, dyslipidemia; DM, diabetes mellitus; EMR, endoscopic mucosal resection; ESD, endoscopic submucosal dissection; GERD, gastroesophageal reflux disease; HT, hypertension; IPMN, intraductal papillary mucinous neoplasm.

### Cases

#### Case 1

**FIGURE 2:**
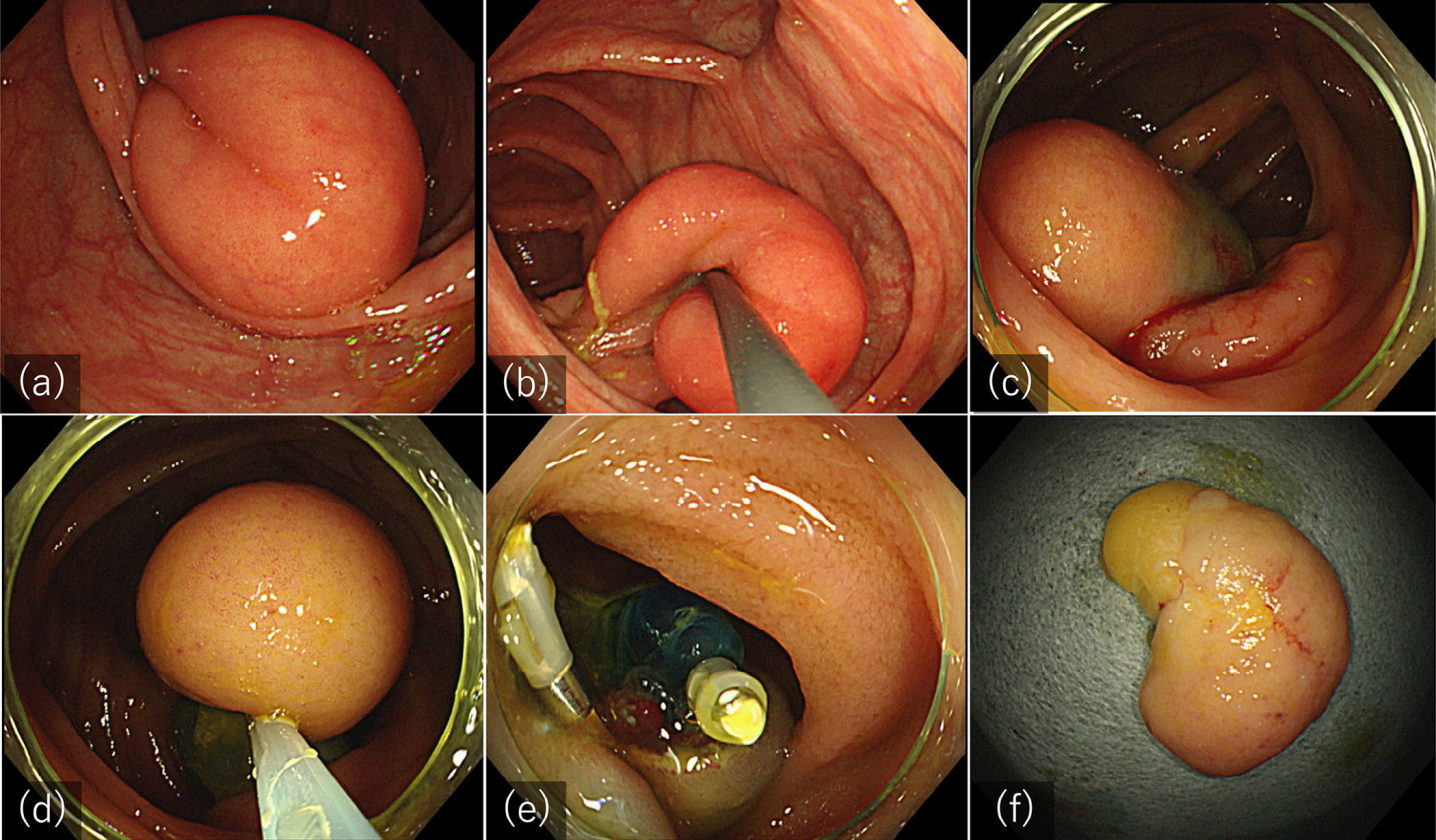
(a) A colonoscopy revealed a 30 mm subepithelial lesion in the cecum. (b) The base of the lesion originated from the ileum, and a diagnosis of terminal ileal lipoma was made. (c) After further pulling out the lesion into the cecum, injection of hyaluronic acid at the base of the lesion stabilized its position. (d) The lesion was resected en bloc by endoscopic mucosal resection. (e) Hemostatic clips were applied on visible vessels. (f) After resection en bloc, a capsulated yellowish lesion was seen under the epithelium.

A 74‐year‐old male with Diabetes Mellitus had a colonoscopy performed for a routine checkup, revealing a yellowish 30mm subepithelial lesion protruding out of the ileocecal valve (Figure 2a). Further examination showed that the base of the lesion was arising from the terminal ileum. The lesion was soft, with a positive cushion sign, and the surface was covered with normal mucosa (Figure 2b). From the above endoscopic findings, the lesion was diagnosed as ileal lipoma. Although asymptomatic, a decision to resect the lesion was made after discussion with the patient.

While insufflation gradually pulled the lesion towards the ileum, the main body of the lesion remained in the cecum. The lesion was further pulled out into the cecum using a snare until the base of the lesion could be observed circumferentially, and the swelling produced by the injection of hyaluronic acid at the base of the lesion secured its position (Figure 2c). Thereafter, the lesion was resected en bloc by EMR using Snaremaster (Olympus) without adverse events (Figure 2d). Visible vessels were closed using hemostatic clips for prophylaxis of delayed bleeding (Figure 2e). The specimen was 30 × 30 × 18 mm, and the histological result showed lipoma (Figure 2f).

#### Case 7

**FIGURE 3:**
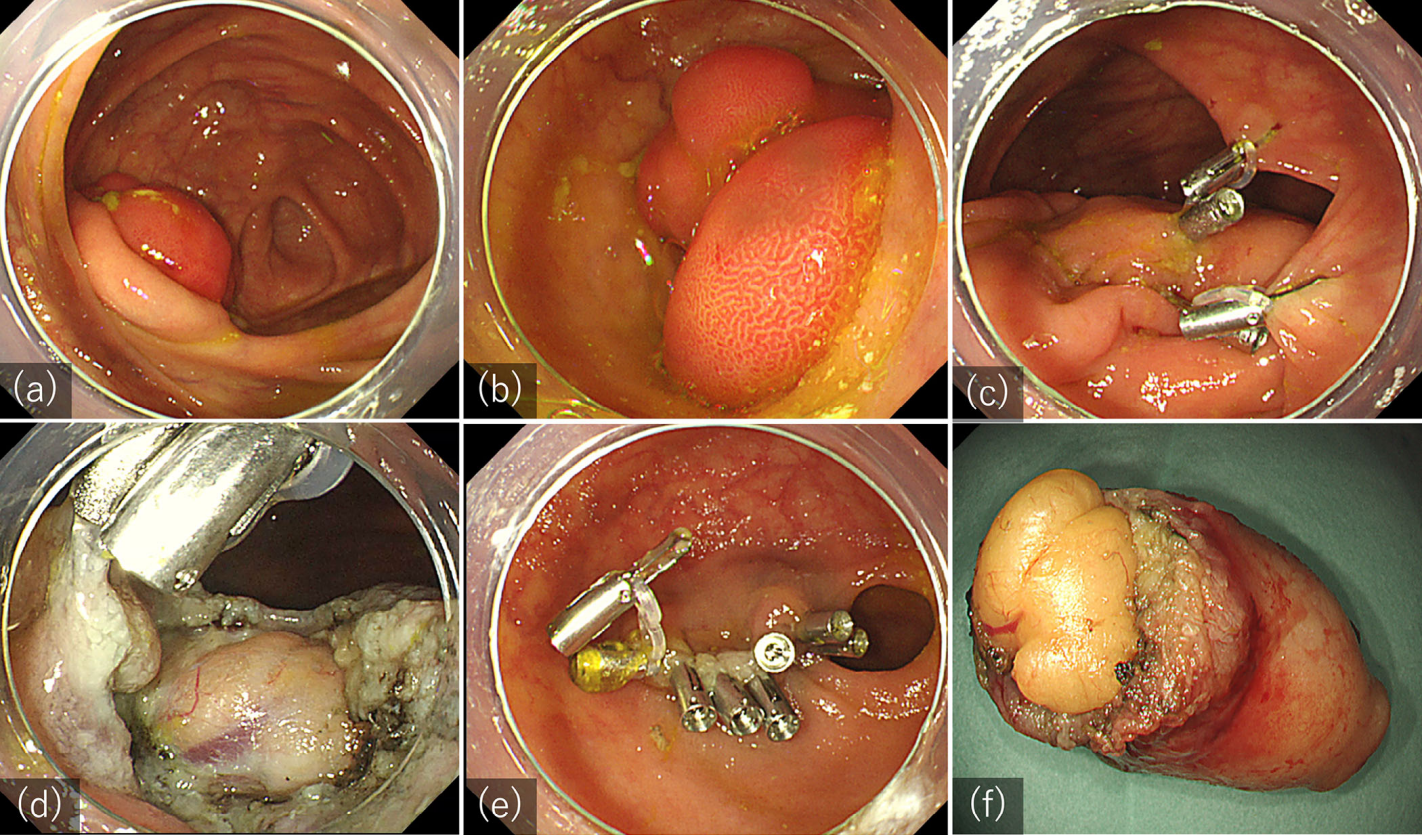
(a) Initially, a 30 mm, reddish, subepithelial lesion was observed, protruding out of the ileocecal valve. (b) Upon insufflation, the lesion was pulled into the ileum, fully occupying the lumen. (c) The lesion was extracted into the cecum and secured in position using clip and band traction between the cecal wall and the base of the lesion. (d) During dissection, the clip and band traction continuously acted as counter traction, enabling visualization of the plane of dissection. A yellowish subepithelial lesion began to appear under the epithelium. An additional elastic traction device was attached to the ileal side of the dissection plane. (e) The post‐endoscopic submucosal dissection defect was fully closed within the cecum using clips, and the traction band was detached for the defect to return inside the ileum. (f) A capsulated yellowish lesion was resected en bloc.

A 53‐year‐old male underwent a routine colonoscopy which revealed a 35 mm, subepithelial lesion at the terminal ileum, protruding from the ileocecal valve (Figure 3a). Upon insufflation, the lesion spontaneously returned to the ileum and was unable to be pulled out by aspiration. Within the ileum, the lesion fully occupied the narrow lumen, making observation of the oral side difficult (Figure 3b). The lesion had a positive cushion sign and the surface epithelium was normal, without erosion or depression. An abdominal computed tomography scan with contrast showed a low attenuation mass protruding into the cecum, and a diagnosis of lipoma was made. Although no symptoms were present, the patient preferred prophylactic treatment by endoscopy. EMR was deemed difficult due to the inability to observe the oral side during snaring, and ESD was performed.

The lesion was initially pulled out of the ileocecal valve using a reopenable clip, under minimal insufflation. An elastic traction device ‐ SureClip traction band (MICRO‐TECH) – which consists of a hemostatic clip with two elastic silicone rings, was then attached to the base of the lesion. The other end of the silicone ring was stretched and clipped onto the cecal wall opposite the ileocecal valve, creating tension between the cecal wall and the lesion. As a result, the lesion was secured in the cecum by the pulling force, enabling visualization of the base. The same procedure was repeated on the other side of the base for stabilization (Figure 3c).

Thereafter, mucosal incision and dissection were done using a Dual Knife (Olympus), from the oral side of the lesion. The clip and band acted as continuous traction, further pulling the lesion into the cecum allowing a better view of the dissection plane (Figure 3d). A soft, yellowish lesion was observed as dissection proceeded (Figure 3d), and the whole lesion was resected en bloc, within 30 minutes, without any adverse events. 

Before the lesion was completely resected, additional traction was applied with an elastic traction device between the cecal wall and the normal ileal mucosa on the oral side, such that the post‐ESD defect would remain in the cecum and not get pulled into the ileum after resection of the lesion (Figure 3d). This allowed clear visualization and hence, a secure and complete closure of the defect (Figure 3e). After closure, the traction band was detached and the defect returned to the ileum. The resected specimen was 34 × 33 × 14 mm, and the histological result showed lipoma without any evidence of adenoma (Figure 3f; Video S1).

## DISCUSSION

Treatment for intestinal lipomas‐ endoscopic or surgical is reserved for symptomatic large lipomas. Intestinal lipomas larger than 2 cm are likely to cause symptoms, but ileal lipomas have the potential to cause symptoms at smaller sizes due to the narrow lumen at the ileum,^1^, ^5^ and treatment can be an option for patients who desire prophylactic resection. Prophylactic resection can especially be considered in larger lesions, as intussusception of such lesions can lead to emergent surgical ileocecal resection. Additionally, as seen in case 5 which had a differential diagnosis of neuroendocrine tumor, resection can also be considered in relatively small lesions when the diagnosis remains uncertain.

Currently, surgical resection remains the mainstay of treatment for ileal lipoma.^1^ Endoscopic treatment in small intestines is difficult, owing to its narrow lumen, thin wall, and abundant mucosal blood supply.^2^ Available endoscopic procedures include EMR, ESD,  “loop‐and‐let‐go”, and unroofing.^3^ Review of previous reports of endoscopic ileal lipoma treatment are shown in the table below (Table 2).

**TABLE 2:** Review of endoscopic treatment case reports for ileal lipomas.

Year	First author	Age (years)	Sex	Symptoms	Size (mm)	Procedure	Procedure site	En bloc resection	Adverse events
1990	T. Matsumoto^13^	72	M	None	30	EMR	N/A	Yes	None
1997	H. Yoshimura^14^	80	F	Abdominal pain	25	EMR	Cecum	Yes	None
2010	T. Morimoto^7^	62	M	hematochezia	50	Unroofing + ESD	Ileum	Yes	Muscle layer laceration
2012	R. Veloso^9^	72	M	Abdominal pain and bloating	16	“Loop‐and‐let‐go”	Ileum	No	None
2013	E. S. Lee^15^	73	F	Abdominal pain and diarrhea	27	EMR	Ileum	Yes	None
2015	A. Ponte^11^	51	F	Abdominal pain and diarrhea	N/A	Endoloop + unroofing	Cecum	No	None
2016	S. B. Javia^16^	67	F	None	20	OTSC + EMR	Ileum	Yes	None
2016	H. Noda^6^	78	F	Abdominal pain	30	ESD	Ileum	Yes	None
2017	M. Ganvir^17^	N/A	N/A	Subacute intestinal obstruction	30	EMR	N/A	N/A	None
2020	H. Zhou^10^	51	F	Bloating	20	Ligation	Ileum	No	None
2020	T. Muramoto^5^	74	M	Abdominal pain	38	ESD	Cecum	Yes	None
2021	A. Teramoto^4^	90	M	intussusception	20	EMR	Ileum	No	None
2021	A. Skamnelos^18^	69	M	+ (Unspecified)	35	Saline immersion ESD	Ileum	Yes	None
2021	H. Y. Chen^2^	44	M	Abdominal pain	40	ESD	Ileum	Yes	None
2022	A. Orhan^19^	58	M	Intussusception	30	EMR	Ileum	Yes	None
2023	K. Cheng^8^	55	F	Abdominal pain and anemia	25	“Loop‐and‐let‐go”	Ileum	No	None
2023	P. Kasapidis^20^	71	F	Intussusception	60	Endoloop + EMR	Cecum	Yes	None

Abbreviations: EMR, endoscopic mucosal resection; ESD, endoscopic submucosal dissection; OTSC, over the scope clip.

Endoscopic mucosal resection using a snare may initially seem simple and safe, but visualization by colonoscopy of the oral side can be difficult in the ileum.^6^ Snaring too deep can lead to perforation, while snaring too shallow may cause difficulty cutting through the electricity‐insulating fat tissue. A previous systematic review of endoscopic resection for colonic lipomas had noted a high rate of perforation in the EMR of lipoma.^3^ “Loop‐and‐let‐go” and unroofing are considered to be a safer method with a low risk of adverse events, but there are reports of incomplete resection leading to recurrence of symptoms.^3^

Endoscopic submucosal dissection has the advantage of being able to resect en bloc,^5^ which is especially essential in cases where the diagnosis remains uncertain. It also holds the advantage of complete resection, eliminating the risk of recurrence of the lipoma and thus the symptoms. However, the technique requires advanced skills and may be associated with the risk of perforation.^2^, ^5^, ^6^, ^12^

We report seven cases of terminal ileal lipomas successfully and effectively resected by extracting the lesion – either spontaneously or endoscopically – into the cecum. From the appearance of the lesion protruding out from the ileocecal valve, we have named this the “tongue out technique” as it resembles a tongue sticking out of a mouth. The procedure can be performed within the cecum, with more working space and a better view. The lesion can either be extracted using forceps, or when the lesion easily returns to the ileum, traction using clips and band/loops can be effective as seen in cases 4, 5, and 7. As larger lipomas may hold malignant potentials or are difficult to distinguish from other malignant lesions,^6^ we utilized endoscopic techniques to resect the lesions en bloc. The technical success rate was 100% and all lesions were resected en bloc. No case of recurrence has been seen to date.

Despite previously published literature suggesting a high risk of perforation with EMR,^3^ EMR was successfully performed in three of our cases with this technique, as the base of the protruding lesion in the cecum could be observed circumferentially, allowing safe snaring. For two lesions with relatively thick stalks, a detachable ligation device was deployed around the base to prevent post‐EMR bleeding. The detachable ligation device was unintentionally trapped within the snare in case 4, which resulted in cutting and subsequent dislodgement of the loop. The resection defect was closed with clips thereafter, but post‐EMR bleeding occurred due to clip dislodgement. The other lesions were resected by ESD, with three recent cases using a traction device to secure the lesion within the cecum. During ESD, incision and dissection were performed from the oral side, causing the lesion to be pulled towards the anal side, enabling better visualization of the dissection plane. 

The biggest advantage of the “tongue out technique” is that, once the lesion is stable inside the cecum, it could be resected in the same manner as any other colonic lesion ‐ a situation that is relatively familiar to endoscopists. Extraction of the lesion will allow a clear circumferential view of the base, hence facilitating ESD and EMR procedures. Using the “tongue out technique”, all seven lesions were resected en bloc without any severe adverse event. Simple and familiar methods can potentially lead to safe and effective procedures, with fewer complications.

This study has several limitations. First, it was a single‐center retrospective study with a small sample size, and as a result, selection bias could not be avoided. The extent of the size of the lipoma that can be safely resected by endoscopic procedure still requires further analysis. Secondly, the distance between the lesion and the ileocecal valve had not been evaluated, and how far the lesion can be located within the ileum for this technique to be applied remains unclear and requires more data. Thirdly, this technique application may be limited to (sub)pedunculated submucosal lesions, as superficial lesions can easily be damaged by the extraction procedure. To our knowledge, there is no previous report of superficial lesions being extracted out of the ileum. On the other hand, this study is the first case series to report on the endoscopic treatment of ileal lipoma. Considering the low prevalence of ileal lipoma, this study is significant for reporting and analyzing seven cases treated with the new technique. The new technique can facilitate treatment of ileal lipomas simply by extracting the lesion out of the ileocecal valve; a technique which can be widely used, requiring no special devices or advanced skills. We achieved technical success in all seven cases, resecting all the lesions en bloc without any severe adverse events. 

In conclusion, extraction to the cecum by “tongue out technique” is a safe and feasible method to perform endoscopic en bloc resection of terminal ileal lipomas. The “tongue out technique” can facilitate the procedure and minimize adverse events, allowing more ileal lipomas to be treated with minimally invasive and organ‐preserving endoscopic procedures.

## CONFLICT OF INTEREST STATEMENT

None.

## Supporting information

VIDEO S1 The ileal lipoma is initially seen protruding out of the ileocecal valve but returns into the ileum upon insufflation. A reopenable clip of the traction device is used to grasp and extract the lesion into the cecum. The appearance of the lesion protruding out of the ileocecal valve resembles that of a tongue sticking out of a mouth. A clip and band traction device is attached to the base of the lesion, and the other end of the band is clipped onto the cecal wall. The pulling force of the traction device secures the lesion within the cecum. The same procedure is repeated on the other side of the base for stability. Mucosal incision and dissection are performed from the oral side of the lesion. Before complete resection of the lesion, additional traction is applied between the normal ileal mucosa and the cecal wall, such that the post‐ESD defect would not get pulled into the ileum. After the resection is completed, the defect is closed using clips. The traction bands are released, and the specimen is collected using a net retriever.

“Tongue out technique” ESD with traction (Supporting Video)
