# Supplementary material for: Extraction of terminal ileal lipomas to cecum can facilitate endoscopic resection: A case series with video
Source: DEN Open. 2024 Apr 30;5(1):e375. doi: 10.1002/deo2.375 (PMC11058687; doi:10.1002/deo2.375)
Supplement: Supplementary file 1 — VIDEO S1 The ileal lipoma is initially seen protruding out of the ileocecal valve but returns into the ileum upon insufflation. A reopenable clip of the traction device is used to grasp and extract the lesion into the cecum. The appearance of the lesion protruding out of the ileocecal valve resembles that of a tongue sticking out of a mouth. A clip and band traction device is attached to the base of the lesion, and the other end of the band is clipped onto the cecal wall. The pulling force of the traction device secures the lesion within the cecum. The same procedure is repeated on the other side of the base for stability. Mucosal incision and dissection are performed from the oral side of the lesion. Before complete resection of the lesion, additional traction is applied between the normal ileal mucosa and the cecal wall, such that the post‐ESD defect would not get pulled into the ileum. After the resection is completed, the defect is closed using clips. The traction bands are released, and the specimen is collected using a net retriever. [file DEO2-5-e375-s002.docx]

【Supplementary Video Legends】

The ileal lipoma is initially seen protruding out of the ileocecal valve but returns into the ileum upon insufflation. Reopenable clip of the traction device is used to grasp and extract the lesion into the cecum. The appearance of the lesion protruding out of the ileocecal valve resembles that of a tongue sticking out of a mouth. A clip and band traction device is attached onto the base of the lesion, and the other end of the band is clipped onto the cecal wall. The pulling force of the traction device secures the lesion within the cecum. Same procedure is repeated on the other side of the base for stability. Mucosal incision and dissection are performed from the oral side of the lesion. Before complete resection of the lesion, additional traction is applied between the normal ileal mucosa and the cecal wall, such that the post ESD defect would not get pulled into the ileum. After the resection is completed, the defect is closed using clips. The traction bands are released, and the specimen is collected using a net retriever.
